# International medical Tourists’ expectations and behavioral intention towards health resorts in Malaysia

**DOI:** 10.1016/j.heliyon.2023.e19721

**Published:** 2023-09-09

**Authors:** Chung Kin Meng, Shishi Kumar Piaralal, Md Aminul Islam, Mohd Faizal Bin Yusof, Rubaiyat Shaimom Chowdhury

**Affiliations:** a Kokoro Development SDN BHD, Malaysia; b Faculty of Business Management, Open University Malaysia, Malaysia; c Faculty of Business & Communication, Universiti Malaysia Perlis, Malaysia; d Faculty of Resilience, Rabdan Academy, United Arab Emirates; e Faculty of Business & Entrepreneurship, Daffodil International University, Bangladesh; f Faculty of Business Administration and Economics, Bangladesh University, Bangladesh

**Keywords:** Medical tourism, International medical tourists, Health resort, Behavioral intentions, Expectations, Malaysia

## Abstract

Medical tourism, a thriving industry encompassing both healthcare and tourism sectors, has experienced exponential growth over the past decades. The intensifying competition within the global market necessitates a closer examination of the pivotal role played by the perceptions of medical tourists in their decision-making process regarding health destination visits. Thus, this study aims to explore the interplay between international medical tourists' perceptions of health resort attractions and their expectations, while also investigating the mediating effect (expectations) of these perceptions on their behavioral intentions to seek medical and healthcare treatments in a Malaysian health resort. Drawing on the Expectation Confirmation Theory (ECT) and the Theory of Planned Behavior (TPB), this research employed a quantitative research method, surveying 386 international medical tourists. The distribution of the survey questionnaire utilized the online internet email method. The study employed the partial least square structural equation modeling (PLS-SEM) method to examine the hypothesized relationships. The results conclusively support the positive influence of health resort attractions on international medical tourists' expectations and behavioral intentions. Consequently, this study provides valuable implications for the future growth and development of the health resort and medical tourism industry in Malaysia.

## 1 Introduction

The roots of medical tourism can be traced back to ancient Greece, where individuals embarked on journeys from across the Mediterranean to Epidauria, seeking healing from Asklepios, the revered god of medicine [1]. Early instances of medical tourism witnessed Europeans flocking to spa towns and sanitoriums to indulge in the therapeutic properties of mineral thermal springs and baths [2]. In the modern era, the concept of medical tourism primarily involved individuals from developing nations traveling to more advanced countries in search of medical services unavailable in their home countries [3]. However, a noteworthy shift has occurred in recent years, giving rise to a new phenomenon known as “reverse medical tourism.” This trend refers to affluent individuals from wealthier nations embarking on medical journeys to developing countries [4]. Motivating factors include the allure of lower healthcare costs and the unavailability of specific medical services in their native lands. Additionally, the decline in quality of medical care and extended waiting periods for treatment have further contributed to this emerging pattern [4].

As global competition in the medical tourism business heats up, medical tourists' opinions become the most important factor in deciding where to get medical care. The cross-border medical-care sector has experienced exponential growth in recent years, with medical tourism development becoming an integral aspect of medical institutions' marketing strategies [5] Specifically, the influence of medical tourists' expectations on their decision-making processes and future actions has been extensively studied. Prior research has consistently demonstrated that medical tourists' views and expectations significantly impact their evaluation of service quality and subsequent behavior within the medical tourism setting [6]. Moreover, perceived quality, perceived value, and satisfaction play a significant role in shaping behavioral intentions towards health resorts [7]. Furthermore, a person's perception, interpretation, imagination, emotions, and overall experience of the care delivery process at a destination greatly influence medical tourism decisions and the intention to revisit [8]. This empirical research study aims to investigate how international medical tourists' expectations of health resort attractions influence their intentions to engage in activities at these destinations. Additionally, the study seeks to explore the sentiment of overseas medical tourists regarding the Malaysian medical care they received. More specifically, this study aims to establish the associations between international medical tourists' perceptions in terms of cost, risk, medical facilities and sustainable tourism, their expectations of health resort attractions, and their behavioral intention of health resorts in Malaysia. Health resort in this study is defined as a health location that provides infrastructure which includes medical facilities, medical treatments, aesthetic services, and sustainable tourism attractions [9].

The researcher adopted the Theory of Planned Behavior (TPB) and the Expectation Confirmation Theory (ECT) as the underlying conceptual framework for this study. By employing these theories, a comprehensive conceptual model was developed to examine the factors influencing the behavior of international medical tourists planning to visit a health resort in Malaysia. Moreover, the study aimed to explore how these relationships are influenced by tourists' expectations. By conducting empirical research, it is expected that the results of the study will showcase how the Theory of Planned Behavior (TPB) and the Expectation Confirmation Theory (ECT theories can be applied to predict the behavioral intentions of international medical tourists who visit a health resort in Malaysia. These findings carry important consequences for prospective health resort ventures, policymakers, and service providers in Malaysia. They offer valuable insights for crafting impactful marketing tactics aimed at enticing a diverse array of medical tourists from various countries. Ultimately, this can contribute to the successful promotion of the health resort and the growth of the medical tourism sector within the nation.

Furthermore, the study's findings may reveal that various factors related to health resort attractions, such as perceived cost, perceived risk, medical facilities, and sustainable tourism, exert a significant influence on the behavioral intentions of international medical tourists. These results can highlight the crucial role of health resort attractions in driving the motivations of international medical tourists to choose Malaysia as their preferred destination. By recognizing and understanding these influential factors, health resorts can enhance their offerings, optimize their services, and align with the preferences and expectations of international medical tourists, thereby increasing their attractiveness and competitiveness in the global medical tourism market.

## 2 Literature review

In recent years, there has been a rapid transformation in the tourism industry as people's interests in health and medical services have become increasingly intertwined [6]. This transformation is driven by individuals' strong desire to improve their well-being and overall quality of life. While medical tourism may differ from traditional vacation travel, it is still considered a form of tourism and has emerged as a thriving industry [10]. Medical tourism refers to the practice of individuals traveling to receive medical treatment, often spanning great distances and crossing borders. These medical tourists seek destinations such as the Caribbean or South America, where they can access medical care while also indulging in leisure activities. People who engage in medical tourism are those who travel for medical care while also pursuing other travel goals, such as leisure, business, or other interests. Lee and Spisto [11] define medical tourism as travel that incorporates medical procedures or activities beneficial to the tourist's health. Another perspective defines medical tourism as an economic activity that integrates two sectors, medicine and tourism, involving the exchange of services [12].

The growth of health tourism can be largely attributed to the increasing popularity of medical travel, which has led to a more diverse and year-round tourist industry [10]. Health tourism encompasses various aspects, including medical tourism and wellness tourism. While wellness tourism focuses on non-medical therapies, medical tourism encompasses a broader range of services.

Within the field of medicine and healthcare, two primary approaches can be identified. The first category comprises all forms of healthcare and medical procedures delivered by licensed medical doctors and dentists [13]. These services encompass traditional medical treatments and interventions. The second group encompasses wellness-oriented medical and healthcare services, which are provided by doctors and other healthcare professionals trained in alternative medicine modalities such as acupuncture, herbal healing, homeopathy, and other non-conventional approaches [6,13]. This distinction highlights the diverse offerings within the realm of medical tourism, catering to the needs and preferences of individuals seeking both traditional medical interventions and alternative therapies for their well-being and health improvement.

Medical tourism refers to the practice of individuals seeking medical care outside their home country, as defined by Ref. [4]. This phenomenon has gained significant attention from researchers, medical service providers, and the impact of social media due to its increasing popularity [9]. Initially, the term “medical tourism” referred to individuals from less-developed countries traveling to developed countries for medical treatments unavailable in their home country [14]. However, in the contemporary context, medical tourism is undergoing both qualitative and quantitative transformations. This shift is characterized by individuals from more developed countries traveling to less developed countries to receive medical care. Several factors contribute to this change, including the relatively lower cost of medical care in less developed countries, the decreasing costs of flights, and the availability of extensive marketing and online information about medical services [14].

The medical tourism industry's potential in Malaysia has been extensively documented, as per Gopalan's [15] research. The promotion of medical tourism in Malaysia was initiated through a dedicated budget allocation, as outlined in the Eight Malaysian Plan (2001–2005) [16] This national plan, formulated by the Malaysian government, encompasses a range of development policies and strategies. The subject in question has persistently garnered both focus and funding throughout the 9th (2006–2020), 10th (2011–2015), and 11th (2016–2020) iterations of the Malaysian Plan. During the budget presentation of 2018, the Prime Minister of Malaysia declared an allocation of 30 million Malaysian Ringgit as a stimulus to augment the medical tourism sector, signifying the government's unwavering backing [17,18].

In addition to the aforementioned factors, it is noteworthy that the Malaysian government has implemented less stringent regulations pertaining to medical advertising, thereby enabling numerous private sector entities to engage in marketing activities both online and offline, within and beyond the country's borders. This initiative was launched by the Malaysian Corporation Healthcare Travel Council (MCHTC) [19], which took over from the National Committee for Promotion of Medical Tourism and Health in 2009. The MCHTC has played a pivotal role in promoting medical tourism in Malaysian hospitals and clinics through a dedicated website [20] that offers access to medical professionals and ancillary services. The present facility in question is primarily under the auspices of a multinational hospital network, with a significant presence in Kuala Lumpur (Selangor), Johor, Malacca, and Penang. According to Sandberg [21], Malaysia was bestowed with the prestigious title of IMTJ Medical Travel Destination of the Year 2015 at the King's Fund in London in September 2015. The Malaysian government's initiatives had a positive impact on Malaysian Medical tourism [22].

### 2.1 Medical tourism in Malaysia

In 1998, the Ministry of Health in Malaysia established the National Committee to promote health tourism and foster the growth of the country's medical tourism industry, as highlighted by Ref. [23]. This initiative aimed to capitalize on the potential economic benefits of medical tourism, such as attracting more tourists and mitigating seasonality issues [24]. Subsequently, in 2009, the Malaysia Travel Council (MHTC) was established to succeed the National Committee as the governing body for international tourism in Malaysia, with the primary objectives of improving healthcare quality and modernizing the healthcare delivery system, and supporting infrastructure [25]. Today, the Malaysia Healthcare Travel Council (MHTC) plays a crucial role in promoting the development of Malaysia's medical tourism industry and positioning Malaysia as a competitive healthcare hub in the Southeast Asian region (ASEAN). As a result of the efforts of MHTC, Malaysia has emerged as a leader in the ASEAN medical tourism market, attracting patients not only from neighboring countries but also from regions such as Indonesia, Singapore, Japan, and West Asia [26]. The number of international patients seeking medical treatment in Malaysia has been steadily increasing over the years. According to data from the Malaysia Healthcare Travel Council (MHTC), there were 641,000 international patients in 2011, 728,800 in 2012, 881,000 in 2013, 882,000 in 2014, 859,000 in 2015, and 921,000 in 2016. The numbers further surpassed a million international patients in both 2017 and 2018 [27]. The revenue generated from medical tourism has also shown consistent growth, with Malaysia earning 527 million Ringgit Malaysia in 2011, 603 million Ringgit Malaysia in 2012, 727 million Ringgit Malaysia in 2013, 777 million Ringgit Malaysia in 2014, 914 million Ringgit Malaysia in 2015, 1123 million Ringgit Malaysia in 2016, 1300 million Ringgit Malaysia in 2017, and 1500 million Ringgit Malaysia in 2018. These figures demonstrate the upward trajectory of medical tourism in Malaysia since 2011. The statistics provided by the Malaysia Healthcare Travel Council (MHTC) encompass a broad range of patients, including experts, migrants, business travelers, tourists, and individuals holding a passport from a country outside their own [27].

Malaysia presents a comprehensive array of medical services that encompass general screening and specialized treatments, including but not limited to cardiothoracic surgery, orthopedics, cosmetic surgery, cancer treatment, rehabilitative medicine, in vitro fertilization, dental treatment, and pain management [28]. Malaysia has emerged as a popular medical tourism destination for a diverse range of countries including Indonesia, middle-eastern countries, China, India, Japan, Australia, and Vietnam [29]. This is because the country's health care is affordable, transportation is easy to use, and getting a visa is quick and easy.

Comprehending the contextual elements of a nation and its society is a crucial aspect for policymakers and practitioners in devising their approaches to augment the competitive edge of Malaysia within the industry [30,31]. Despite the prevalence of country factors in tourism research, the extent to which these factors exert a significant impact on medical tourists who visit Malaysia has yet to be thoroughly investigated [29]. The present study endeavors to direct its attention towards the matter.

### 2.2 Factor affecting medical tourism

Indeed, the growth of medical tourism can be attributed to various factors, as highlighted in prior empirical studies. One significant factor is the increasing demand for healthcare services among individuals seeking medical care [6]. These individuals may be driven by factors such as accessibility, affordability, and quality of medical care. For instance, international medical tourists often prioritize high-quality medical care at a reasonable cost [32]. They may opt for healthcare facilities abroad due to long wait times or limited treatment options in their home countries [13].

Privacy and anonymity are also crucial considerations for medical tourists, particularly those from Western countries who seek plastic surgery or other procedures [4]. The assurance that their medical records and procedures will remain confidential is an important aspect influencing their decision-making process. Additionally, the perception of a destination's safety and stability plays a significant role in attracting medical tourists. Countries with stable governments that ensure the safety of visitors are more likely to be preferred by medical tourists [33]. Strong government support and policies, particularly in the healthcare sector, contribute to the overall appeal of a destination for medical tourism [34]. Few of Asian countries like Thailand, Singapore, and Malaysia, which have demonstrated strong government support in the development of medical tourism, have benefited from this perception [35].

The COVID-19 pandemic has also had a profound impact on health safety considerations. People have become more cautious about cleanliness, hygiene practices, and avoiding crowded places, which has further highlighted the importance of health safety in medical tourism [36]. Asian countries that prioritize health and safety measures are likely to be perceived as attractive destinations for medical tourists in the post-pandemic era. Overall, these factors collectively influence the decision-making process of medical tourists and can contribute to the growth and success of the medical tourism industry in various destinations, including those in Asia.

The phenomenon of medical tourism in Malaysia mainly revolves around private medical institutions, as posited by Rahman [37]. The phenomenon of medical tourism in Malaysia has attracted significant attention as a desirable medical tourism destination in comparison to other ASEAN nations, as evidenced by the works of Pocock [38]. The attractiveness of this phenomenon can be attributed to the presence of competent healthcare professionals, state-of-the-art infrastructure, easily obtainable information and treatment, and aid and backing from governmental bodies and organizations [39]. According to the study conducted by Klijs et al. [36], the majority of patients seeking medical treatment in Malaysia are intra-regional medical travellers. The previously mentioned trends have led to the promotion of medical tourism by neighboring countries, namely Singapore and Thailand. This is primarily due to the perceived economic advantages associated with this sector, as stated by Ebrahim AH [40]. It is noteworthy that Indonesians prefer traveling to Malaysia to receive cardiac treatment, as reported by Saragih HS [41]. Nearly three-quarters of the foreign patients in Malaysia are from Indonesia, primarily from the middle-to upper-income medical strata in Indonesia. According to the research conducted by Md Zain [39], it was observed that a significant proportion of Indonesian medical tourists possessed a relatively high level of education, with as many as 90% having received tertiary-level education. The participants were classified into the middle-upper and high-income categories based on their occupational background. Malaysia's untapped market among Indonesian medical tourists of the upper and middle classes remains promising.

Not only Indonesians, but also medical tourists from China, prefer Malaysia as a destination because of its reasonable prices, convenient transport, and accommodating visa procedures [29]. The attempt to attract and retain Chinese medical tourists has engendered a fierce rivalry. Malaysia is among the numerous stakeholders in the Southeast Asian region. The researcher discovered that Chinese medical tourists exhibit a greater likelihood of positively perceiving Malaysia as a medical tourism destination when they possess knowledge of the country, perceive it as a secure and easily accessible location, and find the medical services to be reasonably priced. Malaysia exhibits a promising potential to emerge as the foremost contender in the medical tourism industry, catering to the diverse requirements of chains of medical tourists.

### 2.3 Factors of health destination choice

The factors mentioned, such as familiarity with the country, safety, and security, medical service costs, accessibility, the influence of social media, quality of medical treatment and services, cost of medical tourism, ease of travel, infrastructure, cultural offerings, visa procedures, and psychological well-being, all play crucial roles in shaping the perception of health destinations and influencing the decisions of medical tourists.

The familiarity with a country and the perception of safety and security are important factors that contribute to the appeal of a health destination [8]. Medical tourists often prefer destinations they are familiar with or perceive as safe and secure, as it instils a sense of trust and comfort.

The cost of medical services and the overall affordability of medical tourism also significantly impact the choice of a health destination [42]. Medical tourists consider the financial aspect of the treatment, including the cost of medical procedures, accommodation, and other related expenses.

Accessibility, including how easy it is to journey and get a visa, is one of the most important factors that bring medical tourists to a certain place. If the process of traveling to a country and obtaining necessary visas is convenient and hassle-free, it positively influences the decision-making of medical tourists [43].

The quality of medical treatment and services offered in a destination, along with the presence of well-developed healthcare infrastructure, are significant factors in attracting medical tourists [42]. The availability of advanced medical facilities, skilled healthcare professionals, and state-of-the-art infrastructure enhances the perceived quality and reliability of a health destination.

Cultural offerings and natural landmarks also contribute to the overall appeal of a health destination [42]. Medical tourists often seek destinations that offer not only medical treatments but also opportunities to explore the local culture, heritage, and natural attractions, making their experience more enriching.

Lastly, the psychological well-being experienced by medical tourists during their stay in the destination country influences their intention to return [44]. Positive patient outcomes, along with overall satisfaction and positive experiences during the medical tourism journey, increase the likelihood of repeat visits and recommendations to others.

Understanding and effectively addressing these factors can help health destinations formulate strategies to attract and retain medical tourists, ultimately contributing to the growth and success of their medical tourism industry.

**Fig. 1:**
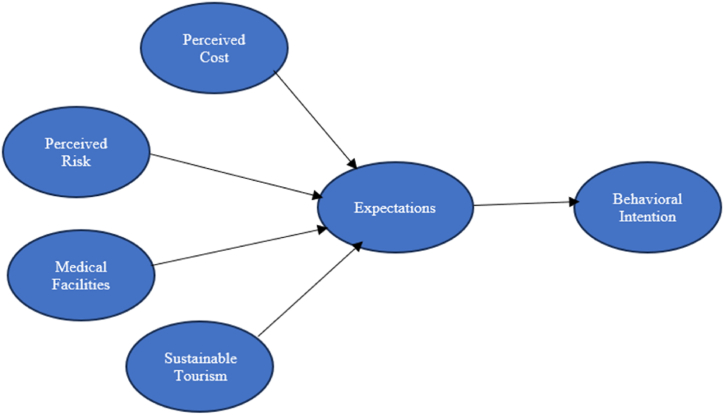
Research framework.

The research framework in Fig. 1 incorporates the Theory of Planned Behavior (TPB), Expectation Confirmation Theory (ECT), and Self-Determination Theory (SDT) to investigate the links between international tourists’ perceptions and their behavioral intentions in the context of health and medical tourism.

The Theory of Planned Behavior (TPB), developed by Ajzen [45], serves as a psychological model for understanding and predicting human behavior based on their intentions and other relevant factors. It posits that behavioral intentions are influenced by three main factors: attitudes towards the behavior, subjective norms (social influences), and perceived behavioral control (perceived ability to perform the behavior). In the context of health and medical tourism, TPB can provide insights into how tourists' perceptions of health resort attractions, costs, risks, medical facilities, sustainability, and quality of care influence their behavioral intentions. Na, Seow & Choong [46], showed that the existing TPB model performs better to explain tourists’ intention to visit Malaysia for medical tourism if the perceived risks, perceived benefits, and resource availability are incorporated with the model.

Expectation Confirmation Theory (ECT), proposed by Oliver [47], focuses on cognitive processes related to confirming or disconfirming expectations about a particular service or experience. In the context of this research, ECT can help understand how tourists’ expectations about the health resort, including the quality of care they expect to receive, mediate the relationship between their perceptions and behavioral intentions.

Self-Determination Theory (SDT), introduced by Deci and Ryan [48], emphasizes the role of autonomy, competence, and relatedness in motivating human behavior. In the context of health and medical tourism, SDT suggests that individuals’ desire for autonomy and control over their healthcare decisions, perceived competence in making informed decisions about medical treatment, and the need for social support and relatedness can influence their inclination to travel overseas for medical treatment.

By integrating these theories, the research aims to explore how tourists’ perceptions of various factors in the health resort context influence their expectations, which, in turn, mediate their behavioral intentions. The TPB provides a framework to understand the underlying factors influencing behavioral intentions, ECT focuses on the role of expectations, and SDT sheds light on the motivational aspects related to autonomy, competence, and relatedness.

Overall, this research framework provides a comprehensive approach to understanding the psychological factors and motivations that shape international tourists’ intentions in the context of health and medical tourism, considering their perceptions, expectations, and the theoretical foundations of TPB, ECT, and SDT.

Fig. 1 depicts the theoretical framework that form the basis of our investigation. The figure presents four arrows symbolizing the logical relationships between different health resort features, namely price, safety, services, and environmental friendliness, and visitor expectations. Additionally, there are four one-way paths originating from health resort attractions, including perceived cost, perceived risk, medical facilities, and sustainable tourism, leading to future activities.

### 2.4 Relationship between cost and medical tourist's expectation

Cost factor is one of the a very crucial issue for many types of business industries which included medical tourism industry. Many prior studies have mentioned that due to the financial consideration of medical tourists, the cost factor in medical tourism industry plays as one of the major roles in measuring behavioral intentions [9,40]. Many researchers have used the cost factor when observing medical tourists’ decision-making processes [9]. Saragih and Jonathan [41] noted that the cost aspect plays an essential role in terms of medical tourism. When individual medical traveller plan for receiving medical treatment services in overseas health destination, cost factor may become the priority consideration issue for such individual. The perceptions of consumers towards products or services are significantly influences their choice behavior. In the aspect of medical treatment services, the cost perceptions of medical tourists frequently affect their decision of choices. Medical tourists utilise these cost perceptions as parameter to evaluate their treatment services and making their attitudes toward the service providers. According to Bagga [14] on the main reason that India has become one of the prominent medical tourism destinations globally is cost factor. The low and affordable medical treatment services cost has made India an attractive healthcare destination among the top medical tourism countries.

H1 Perceived cost of health resort has a significant positive relationship with international medical tourist's expectation.

### 2.5 Relationship between risks and medical tourist's expectation

For patients, medical tourism become necessary when they needed to receive cheaper or better qualities medical treatment services out of their home country [13]. Medical tourists planning for medical travel depended on various combination of factors and research thoroughly on the healthcare methods [33]. There are many reasons behind the decision of medical tourism but the primary concern for the medical tourists is the overall cost of taking medical treatment services in overseas health destination [9]. Medical tourists concerned about whether the overall cost of obtaining the medical treatments in overseas destination is lower than the cost which occurred when receiving treatments in their home country. In the context of medical tourism, the perceived risks retain the significant influence in the medical tourists decision-making process in terms of the selection of health destination. Generally, potential medical tourists have no source to get information related to health destination and medical treatment services. Medical tourist usually depends on medical service providers on online internet to obtain information related to the medical treatment services or health destination [8].

H2 Perceived risk of health resort has a significant positive relationship with international medical tourist's expectation.

### 2.6 Relationship between medical facilities and medical tourist's expectation

Medical tourists in medical tourism usually seek their alternative medical treatments in health destination which facilitated with high standard of medical facilities. In the context of medical tourism, the term medical facilities are not merely meaning to providing advanced medical technology equipment, however it also with the meaning of providing high quality of skilled medical personnel, staffs, and medical services. In order to attract medical tourist to choose certain health destination must fulfil their demand or requirement of high quality advanced medical facilities. In a study which involved medical tourists who visit China has noted that medical facilities and risk levels are significantly influence Indian and Chinese medical travellers’ behaviours [9]. They also argued the findings from their research may help to provide insights related to medical facilities and risk level for the medical service providers to retain their royal customers. Furthermore, these findings also could be applied to the medical tourists from other countries besides India, Thailand, and China. Medical facilities could be considered as one of the main factors that affect medical tourists to decide on travel to certain health destination to receive their medical treatment services.

H3 Medical facilities of health resort has a significant positive relationship with international medical tourist's expectation.

### 2.7 Relationship tourism attraction and medical tourist's expectation

Recently, medical tourism industry has changed the nature of conventional tourism activities speedily. Individual traveller moving across countries to looking for the better and cost-effective medical treatments [9] The term medical tourism is the meaning of both medical and tourism, a combination of two service industries [12]. Medical travellers in medical tourism travel to overseas health destination seeking different types of medical treatment services, such as medical surgeries procedures, dental treatments, etc. While in a stay period in health destination, medical travellers obtaining the medical treatments and at the same time enjoying tourism attractions in order to regain their overall health improvement conditions [6]. Another study by Majeed [9] in their research of exploring key factors of medical tourism and its relation with tourism attraction and re-visit intention indicated that sustainable tourism has a significant positive influence on medical tourists’ behavioral intentions. On the other hand, Majeed et al. [9] in their research noted that the findings pointed out that the behavioral intentions of medical tourists have showed significant positive results when they perceived good sustainable tourism attractions in health destination.

H4 Tourism attractions has a significant positive relationship with international medical tourist's expectation.

### 2.8 Relationship expectations and behavioral intentions

Consumer expectations are stage of beliefs before using product or service. Consumer expectations can be divided into two categories. The first category refers to the predictions of product or service based on past experiences or frequent events, while the second category refers to what consumers prediction based on their ideal or potential imaginations and the information they have received from various sources, such as advertising or expert review. Zolfagharian [22] explained consumer expectations always make consumer to set a range of estimation in mind of product or service about the ideal characteristics before purchase activity. Their expectations of a product or service can be considered as a parameter of their preference for and potential satisfaction towards the product or service. Consumers normally estimate a product or service they have no experience with before purchasing process. Based on the information obtained from external sources for instance, salespersons’ promotion, social media commercial advertisements, or recommendations by others. Many prior studies have pointed out that consumers expectations is the comparison of experiences’ states of products and services which linked to their evaluations of satisfactory levels. The expectations of consumers play an important role in their purchasing behaviours towards products and services. The findings showed that consumers’ expectations has significantly influence consumers’ purchasing intentions. Zolfagharian [22] noted that, in general consumers always form expectations regarding product or service quality before their purchasing behaviours and it is accepted that the consumer expectations will influence their satisfaction and resulting in affect their behavioral choices.

H5 International medical tourist's expectation has a significant positive relationship with behavioral intention.

The primary purpose of employing the Partial Least Squares Structural Equation Modeling (PLS-SEM) is to examine the hypothesized linear relationships between independent variables and dependent variables, both in terms of causation and covariance. PLS-SEM allows for the estimation of path coefficients (β), which indicate the strength and direction of relationships, as well as the evaluation of the model's predictive accuracy (R2) and predictive relevance (Q2). Additionally, the statistical significance and relevance of the path coefficients and effect sizes (f2) will be assessed using PLS-SEM hair [49]. PLS-SEM tends to perform better with smaller sample sizes in compare to CB-SEM [50]. It is more robust when the sample size is relatively small. Therefore PLS-SEM is employed for this study.

## 3 Research methodology

The data collection for this study involved the administration of an online survey, which served as the primary source of data. The survey encompassed a combination of closed-ended questions and scale-response items, strategically incorporated into the questionnaire. The initial section of the survey focused on gathering respondents’ demographic information, employing closed-ended questions. This choice was motivated by the advantages offered by closed-ended questions, such as their simplicity, time efficiency, and the reduced level of expertise required from researchers [51].

The survey questionnaire consisted of seven distinct sections. Section I focused on gathering general information about medical tourists, including their demographic profile. Section II addressed perceived costs, while Section III explored perceived risks associated with medical tourism. In Section IV, participants were asked about their perceptions of medical facilities. Section V delved into the topic of sustainable tourism, while Section VI explored participants' expectations. Finally, Section VII examined participants’ behavioral intentions. This structured questionnaire design allowed for a comprehensive exploration of various aspects related to medical tourism. By dividing the questionnaire into distinct sections, each addressing specific dimensions of interest, the study captured a wide range of relevant data for analysis and interpretation.

In this study, questionnaire for demographic which consist of seven items were self-develop. Medical facilities, perceived risk, perceived cost, sustainable tourism, and behavioral intentions were measured by four items each and whereas expectations were measured by three items had been adapted from Majeed et al. [9] All items chosen in this study are adopted based on the healthcare context and each item were measured using a five-point Likert scales from “strongly disagree” (1) to “strongly agree” (5).

The data analysis for this study have been conducted by using the Structural Equation Modeling (SEM) technique. However, it is important to note that there are no specific guidelines or universally accepted rules for determining the optimal sample size in SEM studies [52]. Nonetheless, researchers such as Hair et al. [49] suggest that a sample size ranging from 150 to 400 is generally considered optimal for SEM investigations. This sample size range is believed to provide accurate and credible results. In our review of relevant literature, we found that many studies in the field of medical tourism have used sample sizes within this range.

Taking these factors into consideration, we have determined that a sample size between 350 and 400 participants will be sufficient to gather the necessary data for this investigation. This sample size range aligns with the recommendations in the literature and ensures an appropriate balance between data comprehensiveness and practical feasibility.

### 3.1 Data collection procedures

The current study relied on an online self-administered questionnaire distributed through the internet by medical travel agents/brokers. The utilization of online self-administered questionnaires not only addressed the challenges associated with confidentiality but also facilitated a more convenient and accessible means of data collection. The involvement of medical travel agents/brokers in Malaysia played a crucial role in recruiting potential volunteers for this study. By working through these agents/brokers, the researcher was able to obtain all the necessary data from the survey questionnaire without directly contacting the respondents. This approach respected the respondents’ right to anonymity.

From mid-June 2021 to mid-July 2021, respondents were reached via online through medical tourism agents/brokers. By utilizing individual medical tourism agents/brokers for recruiting survey respondents, the researcher ensured a sufficient response rate. Additionally, since no identifying information was collected from the responders, their privacy was preserved. This approach guarantees that the data obtained from the survey questionnaire cannot be used to trace or identify the respondents in the future, thus fully protecting their confidentiality and privacy. In this study, participants were subjected to certain preconditions before being allowed to fill out the survey. First, they had to provide informed consent to participate. Second, they must have received medical care in Malaysia previously. Third, they must not be permanent residents of Malaysia and finally at least 18 years old to take part in this study. These preconditions ensured that the participants met the specific criteria necessary for the study and helped maintain the integrity of the data collected.

### 3.2 Data analysis and findings

Preparing for empirical research in the social sciences involves various steps, one of which is coding the data obtained from survey questionnaires. Coding refers to the process of entering the collected information into a computerized statistical analysis program.

In this investigation, a questionnaire consisting of 23 items was used as the measurement scale. A total of 420 questionnaires were distributed to respondents through Internet-based electronic mail. Out of the distributed questionnaires, 402 respondents completed and returned the surveys. The researchers utilized the SPSS statistical software, specifically version 23 to analyze the data. This software was employed to examine the data collected from all the submitted surveys. It facilitated data management, exploration, and statistical analysis. Hair [49] suggested that four steps to counter measure the problems, 1) examine the type of missing data, 2) examine the extent of missing data, 3) examine the randomness of missing data, and finally 4) apply the remedies e.g., imputation method. In this study the researcher utilized two methods to manage the missing date issue. The researcher will use the frequency distributions and descriptive statistics of SPSS statistical software to identifies and screening the data in order to manage the missing data. The purpose of the frequency test is to detect any missing or illegal response for each of the studied variables. Out of the 402 questionnaires received, the researcher determined that 386 questionnaires were useable and suitable for analysis.

## 4 Findings & analysis

### 4.1 Respondents’ demographic characteristics

Out of 402 received questionnaires, the researcher found that 386 useable questionnaires were successfully collected. Table 1 shows how the people who filled out the survey for this research study fit into different groups. Out of 386 people who answered, about 54.4% (210 people) were men and 45.6% (176 people) were women. Most participants were between the ages of 56 and 65 (27.5%), and the second largest age group was between 46 and 55 (27.5%). The rest of the age group included 4.7% of people over 65, 4.4% under 25, 18.3% between 26 and 35, and 18.1% between 36 and 45. (70 respondents). Regarding whether the survey respondents were married, 77.8% (301 respondents) were married, and 17.4% (67 respondents) were not. The rest of the respondents' marital status information was that 2.4% (9 people) were divorced, and 2.4% (9 people) were widowed. Moreover, among the respondents, country of origin statistics show that Australia has the highest representation (29.8%), followed by Indonesia (21.5%), China (20.2%), Singapore (19.9%) and others (8.6%). On the other hand, the educational background of the survey participants showed that 30.5% (117 participants) had a bachelor's degree, 26.3% (101 respondents) had a diploma, and 9.4% (36 respondents) had a postgraduate degree. After that, 22.5% of respondents (89) had at least a high school education, and 11.3% had some schooling (43 respondents).

**Table 1:** Respondents’ demographic data.

Variable	Classification	Frequency	Percentage %
**Gender**	Male	210	54.4
	Female	176	45.6
**Age**	Below 25 years old	17	4.4
	26–35 years old	71	18.3
	36–45 years old	70	18.1
	46–55 years old	104	27
	56–65 years old	106	27.5
	Above 65 years old	18	4.7
**Marital status**	Single	67	17.4
	Married	301	77.8
	Divorce	9	2.4
	Widowed	9	2.4
**Country of Origin**	China	78	20.2
	Australia	115	29.8
	Indonesia	83	21.5
	Singapore	77	19.9
	Others	33	8.6
**Education**	Some school study	43	11.3
	High school	89	22.5
	Diploma	101	26.3
	Degree	117	30.5
	Master degree	36	9.4
**Income (Yearly)**	Less than US25,000	35	9.1
	US 25,001 to US 50,000	117	30.5
	US 50,001 to US 75,000	116	27.5
	US 75,001 to US 100,000	75	21.6
	US 100,000 above	43	11.3
**Employment**	Professional position	55	14.3
	Production/Manufacturing position	47	12.2
	Business Proprietors/Self-employed	116	27.5
	Executive/Management	48	14.9
	Clerical/Administrative/Secretarial	35	9.1
	Retiree/Not in the work force	85	22
**Health tourism**	Within 6 months	117	30.5
**experience in**	Within 1 year	119	30.5
**Malaysia**	Within 2–3 years	67	17.4
	Within 4–5 years	47	12.2
	More than 5 years	36	9.4

When it came to the annual income of the survey participants, 30.5% (117 respondents) made between US$25,001 and US$50,000, 27.5% (116 respondents) made between US$50,001 and US$75,000, and 9.1% (35 respondents) made less than US$25,000.11.3% of respondents made over US$100,000 a year (43 respondents). Most people who answered the survey were business owners or self-employed (27.5%). The second largest group, made up of 85 people, were retired or not working (22%) and made up the second largest group. The rest of the people who answered worked in professional jobs (14.3%, or 55 people), production/manufacturing jobs (12.2%, or 47 people), or executive/management jobs (14.9%). (48 respondents). Most of the people who answered (30.5%, or 119 people) had been to Malaysia for health tourism within the past year. The second largest group, with 30.5% of respondents (117 people), had visited Malaysia in the past six months for health tourism. The rest of the respondents, 17.4% (67 people), 12.2% (47 people), and 9.4% (36 people), went to Malaysia for health tourism within 2–3 years, within 4–5 years, or more than five years.

Table 2 presents means and standard deviations for the Variables, and demonstrates the summary of means and standard deviations for all the variables in this study. For the perceived cost, the result (mean = 4.368, standard deviation = 0.773); perceived risk, the result (mean = 4.412, standard deviation = 0.869); medical facilities, the result (mean = 4.287, standard deviation = 0.729); sustainable tourism, the result (mean = 4.201, standard deviation = 0.737); expectations, the result (mean = 4.482, standard deviation = 0.818); and behavioral intentions, the result (mean = 4.238, standard deviation = 0.801) respectively. Means of all variables were well above the neutral position of 3. These results have presented a strong agreement among the respondents of international medical tourists on all the expressed statements for measured variables in this research. After finishing the descriptive analysis process of variables in this study, the researcher continued with the measurement model analysis in the subsequent section.

**Table 2:** Means and standard deviations for the variables.

Variables	Mean	Standard Deviation
Perceived Cost	4.368	0.773
Perceived Risk	4.412	0.869
Medical Facilities	4.287	0.729
Sustainable Tourism	4.201	0.737
Expectations	4.482	0.818
Behavioral Intentions	4.238	0.801

### 4.2 Measurement and structural model analysis

#### 4.2.1 Internal consistency reliability

In this study, the internal consistency and reliability of the measurement model were assessed using two commonly used measures: composite reliability (CR) and Cronbach's Alpha. The composite reliability (CR) values for all the variables in the measurement model ranged from 0.872 to 0.948, as indicated in Table 3. These values were found to be higher than the recommended threshold of 0.70. According to Nunnally and Bernstein [53], composite reliability values exceeding 0.70 are considered to indicate a high level of reliability. Additionally, all the composite reliability values were below 0.95, which is considered acceptable. Furthermore, the study also examined the results of Cronbach's Alpha, which is commonly used to assess internal consistency. The Cronbach's Alpha values for the constructs in the measurement model ranged from 0.838 to 0.917, as shown in Table 3. Like composite reliability, these values surpassed the threshold of 0.70, indicating a high level of internal consistency and reliability [49]. Both the composite reliability and Cronbach's Alpha results provide evidence that the measurement model used in this study exhibited satisfactory internal consistency and reliability. These findings assure that the variables employed in the model are reliable indicators of the constructs being measured.

**Table 3:** Measurement model results.

Item Level Constructs	Item	LV	CR	α	AVE
	PC1	0.805			
Perceived Cost (PC)	PC2	0.701	0.919	0.813	0.645
	PC3	0.709			
	PC4	0.831			
	PR1	0.836			
Perceived Risk (PR)	PR2	0.717	0.915	0.826	0.632
	PR3	0.791			
	PR4	0.853			
	MF1	0.805			
Medical Facilities (MF)	MF2	0.733	0.817	0.767	0.704
	MF3	0.741			
	MF4	0.829			
	ST1	0.788			
Sustainable Tourism (ST)	ST2	0.763	0.906	0.797	0.609
	ST3	0.805			
	ST4	0.799			
	E1	0.915			
Expectation I	E2	0.877	0.913	0.859	0.784
	E3	0.858			
	BI1	0.821			
Behavioral Intention (BI)	BI2	0.837	0.852	0.822	0.654
	BI3	0.733			
	BI4	0.801			

Note: LV = Loading Value; AVE = Average Variance Extracted; α = Cronbach's alpha; CR = Composite Reliability.

#### 4.2.2 Indicator reliability

In this study, the reliability and validity of the reflection measurement model were evaluated using several criteria, including signal loading, internal consistency reliability, convergent validity, and discriminant validity, as recommended by Hair et al. [49] To assess the reliability and accuracy of the measurement model, the researcher employed confirmatory factor analysis (CFA) within the framework of PLS-SEM. The loading values for all the items associated with each construct in the measurement model were estimated using PLS-SEM, as presented in Table 2. The loading values for the items ranged from 0.725 to 0.938, indicating their significance in relation to their respective constructs. Importantly, all the loading values for each construct surpassed the threshold of 0.70. According to Hair et al. [49], indicator loadings above 0.70 are considered significant and should be retained in the measurement model. These findings suggest that the measurement model used in this study exhibited satisfactory reliability, as indicated by the high loading values. The results support the validity of the model by demonstrating the significance of the indicators in measuring the underlying constructs.

#### 4.2.3 Convergent validity

Convergent validity, as defined by Hair et al. [49] pertains to the extent to which multiple measures of constructs that should theoretically and practically be related are indeed related. In this study, the researcher assessed the convergent validity of the measurement model by examining the correlation coefficients within the PLS-SEM framework. The outcomes of this analysis are presented in Table 3. The average variance extracted (AVE) values for all the constructs in the measurement model were evaluated. These values ranged from 0.673 to 0.858, indicating that each construct accounted for a substantial proportion of the variance shared among its measuring items. Importantly, all the AVE values for the constructs exceeded the recommended threshold of 0.5 [49] The findings demonstrated that the constructs in the measurement model were able to explain more than 50% of the variation observed among the measuring items, thus supporting the convergent validity of the model. The high AVE values provided evidence that the constructs effectively captured the underlying concepts they were intended to measure.

#### 4.2.4 Discriminant validity

A discriminant validity test, as described by Hair et al. [49] assesses the distinctiveness of one variable from others within a study model. In this study, the researcher evaluated the discriminant validity of the measurement model using the Fornell and Larker criterion and the cross-loading method. These approaches examine the relationships between constructs and determine the extent to which one construct differs from others.

Table 4, which presents the results of the discriminant validity analysis based on the Fornell and Larker criterion [54], demonstrates that all the reflective constructs exhibited sufficient discriminant validity. The diagonal entries, representing the square roots of the average variance extracted (AVE), are greater than the off-diagonal entries, representing the correlations between constructs. This indicates that the average square root of each construct's AVE is higher than the correlation with other constructs. Such results signify that the measurement model utilized in this study exhibits discriminant validity. Additionally, Table 5 displays the findings of the Heterotrait-Monotrait Criterion (HTMT) in PLS-SEM. The association values presented in the table are all below 0.85, indicating that all the constructs in the study possess adequate discriminant validity [55].

**Table 4:** Discriminant validity (fornell-larker criterion).

	BI	E	MF	PC	PR	ST
**BI**	0.857					
**E**	0.778	0.926				
**MF**	0.801	0.813	0.852			
**PC**	0.702	0.761	0.762	0.826		
**PR**	0.737	0.784	0.789	0.771	0.829	
**ST**	0.826	0.861	0.805	0.782	0.794	0.827

Note: Diagonal represent the square root of AVE, the off-diagonal represent the correlations.

**Table 5:** Heterotrait-monotrait criterion (HTMT).

	BI	E	MF	PC	PR	ST
BI						
**E**	0.849					
**MF**	0.769	0.837				
**PC**	0.802	0.821	0.822			
**PR**	0.845	0.819	0.761	0.786		
**ST**	0.758	0.843	0.773	0.714	0.793	

Note: BI = Behavioral Intention; E = Expectation; MF = Medical Facilitites; PC = Perceived Cost; PR = Perceived Risk; ST= Sustainable Tourism.

Overall, the outcomes from both the Fornell and Larker criterion and the HTMT analysis support the conclusion that the constructs in the measurement model are distinct from each other, demonstrating satisfactory discriminant validity.

### 4.3 Structural model analysis

In the analysis of the structural model, the researcher aimed to investigate the hypothesized causal relationships between the independent variables and dependent variables. This was accomplished using PLS-SEM, which allowed for the examination of various tests to assess the statistical significance, relevance, effect size, and predictive accuracy of the model. The path coefficient (β) was evaluated to determine the strength and significance of the relationships between the independent and dependent variables. Statistical significance indicates whether the relationship between the variables is unlikely to have occurred by chance. Relevance, on the other hand, assesses the practical significance of the relationships in terms of their magnitude and impact.

The predictive accuracy of the model, measured by R2 (coefficient of determination), provides insights into how well the independent variables explain the variance in the dependent variables. A higher R2 value indicates a stronger predictive power of the model. Additionally, the predictive relevance (Q2) and effect size (f2) of the model were assessed. Q2 indicates the model's ability to predict the dependent variables, while f2 represents the effect size and the strength of the relationships.

**Fig. 2:**
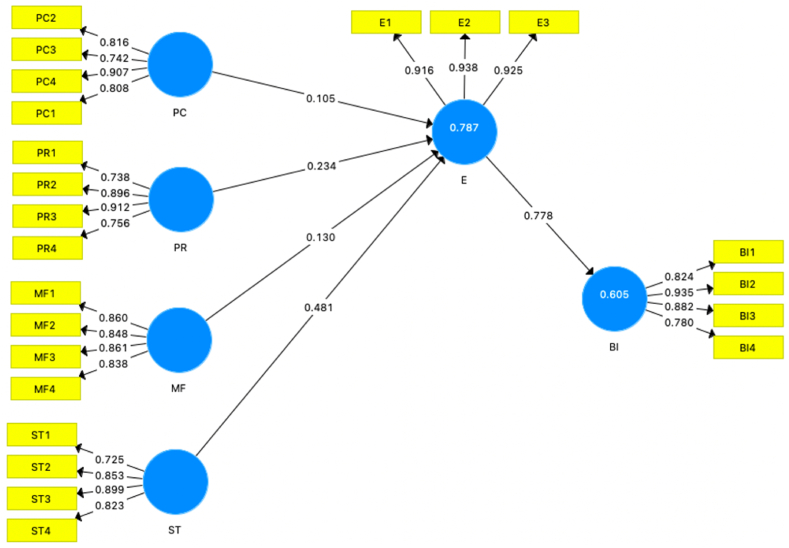
Structural model results.

These tests and measures allow the researcher to comprehensively analyze the structural model, assess the significance and relevance of the relationships, evaluate the predictive accuracy, and understand the overall effectiveness of the model in explaining the causal connections between variables, as outlined in the study by Hair et al. [49]. Fig. 2 shows the structural model results.

The examination of the path coefficient in the structural model allows for the evaluation of the significance and strength of the relationships between predictor constructs. By calculating the path coefficient, the researcher can determine the extent to which the predictor variables influence the outcome variables. The path coefficient analysis is conducted using the Partial Least Squares Structural Equation Modeling (PLS-SEM) technique. This approach enables the examination of the linkages between constructs in the model and provides insights into the direction and magnitude of the relationships.

The main objective of analysing the path coefficients is to assess whether the relationships between the predictor constructs are statistically significant and meaningful. This analysis helps determine whether there is a substantial association between the predictor variables and whether they have a significant relationship with the outcome variables [49].

By examining the path coefficients, researchers can gain a deeper understanding of the relationships within the internal model and determine the strength and direction of these relationships. This information is crucial for evaluating the overall performance of the structural model and understanding the causal links between the predictor and outcome constructs, as described in the study by Hair et al. [49].

The results of the study's path coefficients indicate the strength and significance of the relationships between the latent variables. Fig. 2 and Table 6 present these results. The path coefficients and their corresponding p-values show that there are significant relationships between expectations and behavioral intentions (β = 0.778, p < 0.004), perceived risk (β = 0.234, p < 0.001), medical facilities (β = 0.130, p < 0.000), sustainable tourism (β = 0.481, p < 0.002), and perceived cost (β = 0.105, p < 0.001). These path coefficients provide evidence to support the hypotheses (H1, H2, H3, H4, and H5) proposed in the study. The findings of the structural model suggest that perceived risk, medical facilities, sustainable tourism, and perceived cost have significant impact on expectation and expectations have a significant impact in influencing behavioral intentions, these results contribute to the understanding of the relationships between the variables in the research model and support the theoretical framework of the study.

**Table 6:** Structural model path coefficient.

		β				
H. No.	Path Relations	Standardized Coefficients	p-value	*f* 2	*q*2	Decision
H1	PC → E	0.105	0.001	0.226	0.341	Supported
H2	PR → E	0.234	0.001	0.537	0.426	Supported
H3	MF → E	0.130	0.000	0.285	0.367	Supported
H4	ST → E	0.481	0.002	0.041	0.168	Supported
H5	E → BI	0.778	0.004	0.362	0.283	Supported

Note: BI = Behavioral Intention; E = Expectation; MF = Medical Facilitites; PC = Perceived Cost; PR = Perceived Risk; ST= Sustainable Tourism.

The coefficient (R2) is a measure used to evaluate the predictive ability of a model by indicating the proportion of variance in the dependent variable that can be explained by changes in the independent variables. Higher values of the coefficient (R2) indicate more accurate predictions and a larger amount of explained variance in the latent variables. In this study, the coefficient (R2) values for expectations I and behavioral intentions (BI) were 0.787 and 0.605, respectively, as shown in Table 7. These values suggest that the model has some level of predictive ability and can account for a significant portion of the variance in expectations and behavioral intentions. While the coefficient (R2) values in this study are not extremely high, they still indicate a moderate level of accuracy in predicting the dependent variables based on the independent variables.

**Table 7:** Determination of coefficient.

Item Level Construct	R2
Expectations	0.649
Behavioral Intention (BI)	0.742

Note: R2 = Coefficient of Determination.

The effect size (f2), specifically Cohen's (f2), was employed in this study to assess the magnitude of the causal chain. Cohen's (f2) allows for the comparison of the effect size of each path model. It indicates the increase in the coefficient (R2) associated with the unknown variance of the dependent variable [56]. By examining the (f2) values, the magnitude of the effect of the omitted construct on a given latent or dependent variable can be determined. According to Hair et al. [49], an (f2) value of 0.02, 0.15, or 0.35 corresponds to a minor, medium, or substantial influence, respectively.

In this study, the effect sizes (f2) are presented in Table 4, revealing the magnitude of the impact as follows: Expectations and Perceived Cost (Effect size (f2) = 0.226), Expectations and Perceived Risk (Effect size (f2) = 0.537), Expectations and Medical Facilities (Effect size (f2) = 0.285), and Expectations and Sustainable Tourism (Effect size (f2) = 0.041). These effect sizes indicate medium to large effects. Based on the results of the effect size (f2), all the path models in the structural model demonstrate a strong explanatory power in this study.

The study's structural model underwent a crucial evaluation to assess its predictive capabilities by anticipating future outcomes (Q2). Employing a metaphorical “blindfold,” the researcher meticulously gauged the accuracy of predictions through the recalibration of observed values and parameters within the model, enabling a robust assessment of its predictive potential. As underscored by Hair et al. [49], models are deemed to possess predictive relevance when their prediction relevance (Q2) surpasses zero. Notably, the obtained Prediction relevance (Q2) values for this study were highly notable: Perceived Cost (Q2) = 0.341, Perceived Risk (Q2) = 0.426, Medical Facilities (Q2) = 0.367, Sustainable Tourism (Q2) = 0.168, and Expectations (Q2) = 0.283. Crucially, all the Q2 values pertaining to Prediction relevance ranged between 0.03 and 0.09, signifying their substantial deviation from zero and corroborating the model's remarkable capacity for accurate predictions [49.

## 5 Discussion on findings

### 5.1 Perceived cost and expectations

By reviewing the structural equation model (SEM), the researcher found a significantly positive relationship between international medical tourists' perceived cost and expectations. The study's results revealed a significant positive relationship between the perceived cost and expectations of international medical tourists in the context of health resorts in Malaysia, as indicated by the significant standardized path coefficient (β = 0.169, p < 0.005). It was observed that meeting the perceived cost demands of international medical tourists positively influenced their expectations. Therefore, addressing the cost perspective is crucial in influencing their expectations and generating favorable decision-making processes in the realm of medical tourism.

The significance of perceived cost in shaping consumer behavior has been recognized in prior literature [9,32]. When it comes to medical tourism, how much people think something will cost has a big effect on how happy they are and what decisions they make. Previous studies have shown that consumer perception of the price of a product or service is an important factor in the purchase decision. As discussed in earlier chapters, the level of cost or price expectations for medical services is essential for international medical tourists when forming their positive or negative behavioral intentions [32]. Meeting medical tourists’ expectations regarding perceived cost can lead to positive behavioral intentions. Therefore, the findings of this study can serve as guidance for health resorts involved in medical tourism, helping them enhance the positive perception of cost in essential medical service attributes during the delivery process and simultaneously improve the level of expectations among medical tourists.

### 5.2 Perceived risk and expectations

This study examines the relationship between international medical tourists’ perceived risk and their expectations in the context of health resorts in Malaysia. The results demonstrate a significant effect of perceived risk on the expectations of international medical tourists (β = 0.327, p < 0.05). The finding suggests that reducing the level of perceived risk can positively influence expectations and ultimately have a constructive impact on the intentions of international medical tourists to visit a health resort in Malaysia.

These findings align with prior studies that have also identified a positive relationship between perceived risk and expectations in the context of medical tourism [9]. This further validates the positive influence of perceived risk on the expectations of international medical tourists in the context of medical tourism. Perceived risk has been identified as a crucial factor in medical travelers' decision-making attitudes, as highlighted by Seow [34]. How risky tourists think their trips are can have a big effect on their choices. Most of the time, medical travelers don't go to foreign medical destinations if they think the risk is too high.

Therefore, the findings of this study emphasize the importance of addressing and mitigating perceived risk in the medical tourism context. By reducing perceived risk, health resorts and medical tourism providers can positively shape the expectations of international medical tourists and enhance their intentions to visit Malaysia for medical purposes.

### 5.3 Medical facilities and expectations

The analysis of the gathered data in this study reveals a positive direct relationship between medical facilities and the expectations of international medical tourists in the context of health resorts in Malaysia. The significant standardized path estimate (β = 0.389, p < 0.05) indicates that there is a substantial positive association between medical facilities and the expectations of international medical tourists in the health resort context.

In the medical tourism industry, medical facilities play a crucial role in influencing the travel decisions of medical travelers. The quality and availability of medical facilities can significantly impact the expectations and satisfaction of medical tourists. In the medical tourism business, satisfaction is a key sign of success. Furthermore, expectations play a key role in the medical tourism industry as they contribute to the satisfaction of medical tourists who seek to achieve their health goals through health service providers in medical destinations [35].

Numerous prior studies have also highlighted the influence of medical facilities on expectations and subsequent satisfaction of international medical tourists in the context of medical tourism. The quality of medical facilities directly affects the expectations of medical tourists, leading to their satisfaction. Satisfaction, in turn, plays a significant role in shaping the positive behavioral intentions of international medical tourists in the chosen health destination [6].

Therefore, the findings of this study underscore the importance of medical facilities in health destinations. If the features and quality of medical facilities can generate positive expectations among international medical tourists, it is likely to result in their satisfaction and positively influence their behavioral intentions in the health resort context. Enhancing and promoting high-quality medical facilities can thus contribute to the success and attractiveness of a health resort in the medical tourism industry.

### 5.4 Sustainable tourism and expectations

The objective of this study was to examine the relationship between sustainable tourism and the expectations of international medical tourists in the context of health resorts. The statistical analysis conducted in the previous chapter yielded a significant standardized path estimate (β = 0.119, p < 0.05). This finding indicates a significant positive relationship between sustainable tourism and the expectations of international medical tourists in the perspective of health resorts in Malaysia.

The result suggests that a higher level of sustainable tourism in a health destination is likely to attract more medical tourists. This implies that offering sustainable tourism experiences before or after medical treatments can make a medical destination more desirable for medical tourists. Therefore, the findings of this study align with previous literature that has emphasized the significant role of sustainable tourism in shaping positive expectations among international medical tourists [9,13].

In conclusion, the results of this study highlight the importance of sustainable tourism in the context of health resorts and its impact on the expectations of international medical tourists. Health destinations that prioritize sustainable tourism practices can enhance their appeal and attract more medical tourists. By offering sustainable tourism experiences alongside medical treatments, health resorts can create a more desirable and well-rounded destination for international medical tourists.

### 5.5 Expectations and behavioral intentions

The relationship between international medical tourists' expectations and their behavioral intentions was examined in this study, and the analysis revealed a significant standardized path estimate (β = 0.295, p < 0.05). This finding indicates a significant positive relationship between international medical tourists’ expectations and their behavioral intentions. Specifically, when the level of expectations among international medical tourists is higher, it positively influences their behavioral intentions, such as their intention to visit a health resort in Malaysia.

Enhancing the expectations of international medical tourists towards health resorts in Malaysia can therefore increase their intention to visit such destinations. This finding aligns with previous literature that highlights the importance of expectation confirmation in consumer purchasing behavior. Numerous empirical studies in the context of consumer behavior have shown that consumers' expectations of products or services are positively related to their purchase intentions. The level of purchase intentions is influenced by the extent of their expectations [57]. In other words, when consumers’ expectations of products or services increase, their likelihood of engaging in transactions or purchasing those products or services also increases.

In the healthcare industry, research has demonstrated that healthcare consumers tend to be satisfied when their expectations regarding the quality of a product or service are exceeded [58]. This satisfaction leads to positive behavioral intentions. Similarly, in the context of medical tourism, when the expectations of international medical tourists are met or exceeded by health resorts in terms of quality and service, it can result in increased satisfaction and ultimately lead to positive behavioral intentions, such as their intention to visit the health resort.

Overall, the findings of this study support the idea that international medical tourists’ expectations have a significant impact on their behavioral intentions. By understanding and meeting the expectations of international medical tourists, health resorts in Malaysia can enhance their appeal and attract more medical tourists, leading to positive outcomes for both the tourists and the health resorts.

## 6 Conclusion

The growth of medical tourism in the Asia-Pacific region, including Malaysia, has been significant over the past decade. Unlike other forms of tourism, medical tourism involves individuals traveling for medical purposes alongside their business activities. Malaysia has emerged as a prominent destination in the medical tourism industry and is actively expanding its offerings to attract more international medical tourists. To maintain its position and stay ahead of the competition, Malaysia's medical tourism sector faces the challenge of encouraging positive behavior among international medical tourists and enticing them to choose Malaysia as their preferred treatment destination. This requires the development of innovative marketing strategies by service providers in the medical tourism industry. The findings of this study can contribute to the advancement of the medical tourism sector in Malaysia by shedding light on the factors that influence the behavior of international medical tourists. By understanding these factors, medical service providers can tailor their marketing strategies to meet the needs and expectations of potential medical tourists. This knowledge can be used to attract more medical tourists and differentiate Malaysia's offerings from other competing destinations. In conclusion, the findings of this study can serve as a valuable resource for Malaysian medical service providers in designing effective marketing strategies. By leveraging the insights gained from this research, the medical tourism sector in Malaysia can enhance its competitiveness and attract a larger share of international medical tourists.

### 6.1 Theoretical contribution & implications

This study aimed to address the gap in health resort literature from the perspective of international medical tourists visiting Malaysia. The researcher utilized the Theory of Planned Behavior (TPB), Expectation Confirmation Theory (ECT), and Self-Determination Theory (SDT) as the theoretical foundations to develop the conceptual framework for the study.

The conceptual framework Incorporated four independent variables, which were the attractions of the health resort: Perceived Cost, Perceived Risk, Medical Facilities, and Sustainable Tourism. Expectations were considered as the mediating variable, while Behavioral Intentions served as the dependent variable.

The findings of the study have significant implications for marketing theory. The factors related to health resort attractions were found to play crucial roles in influencing the behavioral intentions of international medical tourists. This suggests that the appeal of health resorts, including factors such as perceived cost, perceived risk, medical facilities, and sustainable tourism, are vital in attracting the visit intentions of international medical tourists to health resorts in Malaysia. Meeting the expectations of international medical tourists through the consideration of these health resort attractions is likely to increase their visit intentions.

The study contributes valuable insights to the understanding of medical tourist behavior towards health resorts, particularly in the context of Malaysia. It specifically addresses medical tourists' visit intentions towards health resorts in Malaysia, providing new knowledge and implications for medical tourism studies. Moreover, the empirical research conducted in this study focused on actual international medical tourists who had experienced medical treatments in Malaysia, which is a unique contribution. Previous quantitative research in this field mostly focused on potential medical tourists, making this study's insights particularly meaningful and relevant to understanding the behavioral intentions of actual medical tourists.

### 6.2 Managerial implications

From a managerial perspective, the independent variables of health resort attractions, including perceived cost, perceived risk, medical facilities, and sustainable tourism, along with the mediating variable of expectations, have been shown to significantly predict the intentions of international medical tourists. Therefore, it is crucial for health resorts in Malaysia to cultivate positive perceptions and favorable experiences related to these attractions in order to attract and retain international medical tourists. The findings emphasize the importance of creating a positive perception of health resort attractions to motivate international medical tourists to choose Malaysia as their destination. This implies that health resort developers and management teams should invest in enhancing the quality and appeal of these attractions. By understanding and addressing the factors that influence international medical tourists' expectations, health resorts can effectively shape their visitors’ behavioral intentions. The significant impact of health resort attractions on expectations and subsequent behavioral intentions provides valuable insights for future health resort development and promotion. It highlights the need for health resort service providers, including developers and management teams, to fully comprehend the significance of health resort attractions and how they can be strategically shaped and improved.

A crucial aspect to consider is the perceived cost of the health resort experience, as it plays a key role in setting appropriate pricing policies that meet the cost requirements of medical tourists. Pricing policies should be implemented with a focus on offering reasonable and fair prices, ensuring that the cost remains affordable for the targeted medical tourists. Promoting clear price information, including highlighting competitive pricing compared to other regional competitors, can help achieve this goal. In addition, service providers should be mindful of the perceived risks that international medical tourists may face. Understanding the different types of perceived risks and taking proactive measures to address them can enhance safety perception and instill confidence in medical tourists before and after their visit to the health resort.

The medical facilities within the health resort should be designed to meet the specific requirements of international medical tourists. This includes providing quality medical services, advanced and modern medical equipment, as well as recreational and safety facilities. By incorporating these elements into the development of the health resort, a comprehensive and satisfying experience can be created for medical tourists. To promote sustainable tourism, service providers should collaborate closely with local travel agencies to develop tourism products and activities tailored to medical tourists, such as sightseeing and shopping tours. This ensures that medical tourists have a holistic and enjoyable experience before and after their treatments, contributing to the overall satisfaction and ease of their medical tourism in the health destination. Furthermore, integrating relaxation and recreation activities within the health resort can enhance the overall experience for medical tourists, allowing them to feel a sense of relaxation and enjoyment during their stay.

Overall, the study's findings suggest that a focus on enhancing health resort attractions and meeting the expectations of international medical tourists is essential for the success of health resorts in Malaysia. By understanding and addressing these factors, health resort managers and developers can better position themselves to attract and satisfy international medical tourists, ultimately contributing to the growth and competitiveness of the medical tourism industry in Malaysia.

### 6.3 Policy implications

The findings of this study can serve as valuable references for policymakers in Malaysia to develop appropriate policies and effective marketing strategies that promote the growth of the medical tourism industry. One potential policy approach is to introduce tax incentive schemes that support health resort service providers, enabling them to incorporate the advantages identified in this study into their pricing policies and offer more competitive prices. By doing so, service providers can better meet the cost requirements of international medical tourists, making the health resort sector more appealing to them.

In addition, policymakers could consider providing financial support or loan programs specifically designed for health resort service providers. This support would enable them to upgrade their medical facilities without concerns about their operational capital. By assisting service providers in acquiring advanced and modern medical equipment, the policymakers would contribute to enhancing the visit intentions of international medical tourists. Upgraded facilities would not only attract more medical tourists but also improve the overall quality of healthcare services provided.

By offering these supports, policymakers in Malaysia would not only promote the development of health resorts but also stimulate the growth of the medical tourism industry. This approach would establish Malaysia as an attractive destination for international medical tourists, benefiting both the health resort sector and the country's economy.

Given that the study highlights the significant influence of perceived risk on the behavioral intentions of international medical tourists in the context of health resorts in Malaysia, it is recommended that the Malaysian Government, particularly the Ministry of Health, consider implementing medical regulation policies to safeguard the interests of medical tourists. Safety and the quality of medical services are paramount concerns for medical tourists, and addressing these concerns through regulatory measures can instil a sense of security and confidence in their decision to choose Malaysia as a medical tourism destination.

By implementing laws and regulations that protect international medical tourists, the Ministry of Health can effectively ensure their safety and well-being throughout their stay in the country. Such regulations can cover various aspects, including accreditation and certification of healthcare facilities, standards for medical procedures, licensing of healthcare professionals, and guidelines for ethical practices. Clear regulations and guidelines will contribute to improving the overall quality and reliability of healthcare services provided to international medical tourists.

By establishing and enforcing these regulations, the Ministry of Health can foster a safe and protected environment for medical tourists, thereby enhancing their trust in the healthcare system of Malaysia. This, in turn, can attract more international medical tourists to not only visit health resorts in Malaysia but also benefit the entire medical tourism industry in the country. International recognition of Malaysia as a destination with robust medical regulations will contribute to building a positive reputation and positioning the country as a preferred choice for medical tourism.

It is important for the Ministry of Health to collaborate with relevant stakeholders, including health resort service providers, medical professionals, and industry associations, to develop comprehensive and effective regulatory policies. Regular monitoring, evaluation, and continuous improvement of these policies will be essential to ensure their effectiveness and alignment with international standards.

By prioritizing the implementation of medical regulation policies, the Ministry of Health can demonstrate its commitment to the well-being and satisfaction of international medical tourists, bolstering Malaysia's position as a leading destination for medical tourism.

### 6.4 Limitations and future research directions

This study provides valuable insights into health tourism at health resorts, but it has also limitations. The cultural background of the sampled population, which consisted of international medical tourists, affects their perceptions and intentions. Future research should consider cultural influences on international medical tourists’ behavior.

Another limitation is the focus on specific attractions of health resorts including additional factors like patient-friendly accommodations and alternative therapies would improve understanding of international medical tourists’ intentions.

The sampling population is also limited to international tourists with previous medical treatment experiences in Malaysia. Future studies should include those without treatment experiences to explore factors influencing medical tourism expectations. Additionally, including local Malaysian medical tourists would offer insights into their perceptions and intentions, allowing health resort providers to better target local consumers.

## Author contribution statement

Chung Kin Meng: conceived and designed the experiments; wrote the paper.

Shishi Kumar Piaralal: performed the experiments; contributed reagents, materials, analysis tools or data;

Md. Aminul Islam: conceived and designed the experiments; analyzed and interpreted the data.

Md. Faizal Bin Yusof: performed the experiments; contributed reagents, materials, analysis tools or data; Rubaiyat Shaimom Chowdhury: analyzed and interpreted the data; wrote the paper.

## Funding statement

The APC is funded by the Rabdan Academy, United Arab Emirates.

## Data availability statement

The data that has been used is confidential.

## Declaration of interest's statement

The authors declare no conflict of interest.

## Declaration of competing interest

The authors declare that they have no known competing financial interests or personal relationships that could have appeared to influence the work reported in this paper.
