# Supplementary material for: International medical Tourists’ expectations and behavioral intention towards health resorts in Malaysia
Source: Heliyon. 2023 Sep 9;9(9):e19721. doi: 10.1016/j.heliyon.2023.e19721 (PMC10559008; doi:10.1016/j.heliyon.2023.e19721)
Supplement: Multimedia component 1 [file mmc1.docx]

**COVER LETTER**

Dear Sir/Madam

Re: **Survey Research Questionnaires** **– To Measure the Behavioural Intentions of International Medical Tourists of Health Resorts in Malaysia**

In reference to the above, I am a post-graduate student of Doctor Business Administration and would like to seek your support and corporation in completing the research survey. This survey is conducted as part of the requirement to fulfil the partial requirement of the degree of Doctor in Business Administration at the Open University Malaysia, Kuala Lumpur.

The survey is to examine the behavioural intentions of international medical tourists on health resorts in Malaysia. The objective of this study is with the attempts to provide some understanding to the factors that determining the visit intentions of international medical tourists on health resorts in Malaysia.

Along with this letter, there is a short questionnaire for you to answer. Instructions for completing the questionnaire can be found on the form itself. It is very much appreciated if you could complete and return the questionnaire as soon as possible. It should take you about 15 minutes to complete the questionnaire.

Please do not state your name on the questionnaire form. Any of your responses will not be identified with you personally. The results of this survey will be treated as private and confidential information and are only be used for academic research purposes. Lastly, the researcher will appreciate and thank you for your cooperation and help to complete this survey.

Your Sincerely;

Chung Kin Meng

DBA student

Open University Malaysia

**SURVEY QUESTIONNAIRE**

**Section A: General Information**

Please tick (✓) on the appropriate box or fill in the

1. What is your gender?
 ( ) Male ( ) Female

2. What is your marital status?

( ) Single ( ) Married ( ) Divorced ( ) Widowed

3. What is your age group?

( ) 25yearsoldandbelow ( ) 26–35yearsold ( ) 36–45yearsold

( ) 46–55yearsold ( ) 56–65yearsold ( )above65yearsold

4. What is your highest educational level?

( )  High school or below ( )  Certificate or Diploma ( )  Professional certificate

( ) Bachelor’s degree ( ) Postgraduate education

5. Which one of the following best describes your employment?

(  ) Professional position (  ) Production/Manufacturing position (  ) Business Proprietors/Self-employed

(  ) Unemployed ( ) Executive/Managerial position

( ) Clerical/Administrative/Secretarial ( ) Retiree/Not in the work force

6. What is your net income per year?

( ) Below USD 25 thousand ( ) Between USD 25,001 to 75,000

( ) Between USD 75,001 to 100,00 ( ) Above USD100,000

7. What is your experience of health tourism in Malaysia?

( ) Within 6 months ( ) Within 1 year ( ) Within 2 - 3 years

( ) Within 4 – 5 years ( ) More than 5 years

| **Section B: Survey Questionnaire Items** | | |  | | |  | | | |  | | |  | | |  | | |  |
| --- | --- | --- | --- | --- | --- | --- | --- | --- | --- | --- | --- | --- | --- | --- | --- | --- | --- | --- | --- |
|  | |  | | |  | | |  | | |  | | | |  | |  | | |
| **No.** | **Contents** | | |  | | |  | | **Agreement** | | | | |  | | | |  |  |
|  | **Perceived Cost** | | | **Strongly Disagree** | | | **Disagree** | | **Neutral** | | | **Agree** | | **Strongly Agree** | | | |  |  |
| 1 | I will prefer to save the time/effort required to access different medical/healthcare/aesthetic services and lodging facility | | | 1 | | | 2 | | 3 | | | 4 | | 5 | | | |  |  |
| 2 | I will choose to find my favoured healthcare/medical treatment economically than other healthcare/medical settings. | | | 1 | | | 2 | | 3 | | | 4 | | 5 | | | |  |  |
| 3 | I will favour to travel easily from my country at a lower cost. | | | 1 | | | 2 | | 3 | | | 4 | | 5 | | | |  |  |
| 4 | I will prefer to use health/medical/wellness services with favourable exchange rates. | | | 1 | | | 2 | | 3 | | | 4 | | 5 | | | |  |  |
|  |  | | |  | | |  | |  | | |  | |  | | | |  |  |
|  | **Perceived Risk** | | |  | | |  | |  | | |  | |  | | | |  |  |
| 5 | I will wish to avoid the risk that the intended healthcare/medical treatment will not produce the desired results as expected. | | | 1 | | | 2 | | 3 | | | 4 | | 5 | | | |  |  |
| 6 | I will prefer to avoid the risk that existing health condition will be worst if I travel to another destination. | | | 1 | | | 2 | | 3 | | | 4 | | 5 | | | |  |  |
| 7 | I will favour to avoid the risk that my healthcare expenses, overall cost which will not bring benefit to me. | | | 1 | | | 2 | | 3 | | | 4 | | 5 | | | |  |  |
| 8 | I will prefer to avoid the risk of accidental injury or any kind of possible danger on me. | | | 1 | | | 2 | | 3 | | | 4 | | 5 | | | |  |  |
|  |  | | |  | | |  | |  | | |  | |  | | | |  |  |
|  | **Medical Facilities** | | |  | | |  | |  | | |  | |  | | | |  |  |
| 9 | I will prefer to find technically advanced medical facilities. | | | 1 | | | 2 | | 3 | | | 4 | | 5 | | | |  |  |
| 10 | I will favour to find good aesthetic services (e.g., alternative treatments, diet and exercise plan etc.). | | | 1 | | | 2 | | 3 | | | 4 | | 5 | | | |  |  |
| 11 | I will choose to find a quality post-operative care with privacy and confidentiality. | | | 1 | | | 2 | | 3 | | | 4 | | 5 | | | |  |  |
| 12 | I will prefer to find the services of qualified medical staff. | | | 1 | | | 2 | | 3 | | | 4 | | 5 | | | |  |  |
|  |  | | |  | | |  | |  | | |  | |  | | | |  |  |
|  | **Sustainable Tourism** | | |  | | |  | |  | | |  | |  | | | |  |  |
| 13 | I will favour to relax mentally and physically in non-polluted/less polluted sites | | | 1 | | | 2 | | 3 | | | 4 | | 5 | | | |  |  |
| 14 | I will like to experience the beauty of nature, e.g., beaches, and fun. | | | 1 | | | 2 | | 3 | | | 4 | | 5 | | | |  |  |
| 15 | I will prefer to find a package that offers healthcare/medical treatment and tourism adventure to lodging with good meal. | | | 1 | | | 2 | | 3 | | | 4 | | 5 | | | |  |  |
| 16 | I will prefer to find the feeling of well-being to me. | | | 1 | | | 2 | | 3 | | | 4 | | 5 | | | |  |  |
|  |  | | |  | | |  | |  | | |  | |  | | | |  |  |
|  | **Expectations** | | |  | | |  | |  | | |  | |  | | | |  |  |
| 17 | I expect that a health resort will help me with a good health/medical/tourism experience. | | | 1 | | | 2 | | 3 | | | 4 | | 5 | | | |  |  |
| 18 | A health resort would provide better medical/healthcare care than I expect. | | | 1 | | | 2 | | 3 | | | 4 | | 5 | | | |  |  |
| 19 | I expect a health resort will offer a valuable care than the care available in other healthcare/medical locations/hospitals etc. | | | 1 | | | 2 | | 3 | | | 4 | | 5 | | | |  |  |
|  |  | | |  | | |  | |  | | |  | |  | | | |  |  |
|  | **Behavioural Intentions** | | |  | | |  | |  | | |  | |  | | | |  |  |
| 20 | I will prefer to visit a health resort while traveling in another country for health care and medical treatment. | | | 1 | | | 2 | | 3 | | | 4 | | 5 | | | |  |  |
| 21 | I will prefer a health resort for my healthcare and medical treatment. | | | 1 | | | 2 | | 3 | | | 4 | | 5 | | | |  |  |
| 22 | I will tell others about my experience in the health resort. | | | 1 | | | 2 | | 3 | | | 4 | | 5 | | | |  |  |
| 23 | I will prefer to find my health treatments in health resort than stand-alone facilities. | | | 1 | | | 2 | | 3 | | | 4 | | 5 | | | |  |  |
